# Effects of the Fruit Extract of* Tribulus terrestris* on Skin Inflammation in Mice with Oxazolone-Induced Atopic Dermatitis through Regulation of Calcium Channels, Orai-1 and TRPV3, and Mast Cell Activation

**DOI:** 10.1155/2017/8312946

**Published:** 2017-11-14

**Authors:** Seok Yong Kang, Hyo Won Jung, Joo Hyun Nam, Woo Kyung Kim, Jong-Seong Kang, Young-Ho Kim, Cheong-Weon Cho, Chong Woon Cho, Yong-Ki Park, Hyo Sang Bae

**Affiliations:** ^1^Department of Herbology, College of Korean Medicine, Dongguk University, 123 Dongdae-ro, Gyeongju 38066, Republic of Korea; ^2^Department of Physiology, College of Medicine, Dongguk University, Dongdae-ro 123, Gyeongju 38066, Republic of Korea; ^3^Department of Internal Medicine, Graduate School of Medicine, Dongguk University, Dongguk-ro 27, Ilsan Dong-gu, Goyang, Gyeonggi-do 10326, Republic of Korea; ^4^College of Pharmacy and Institute of Drug Research and Development, Chungnam National University, Daejeon, Republic of Korea; ^5^Department of Sasang Constitutional Medicine, College of Korean Medicine, Dongguk University, 27 Dongguk-ro, Ilsan Dong-gu, Goyang, Gyeonggi-do 10326, Republic of Korea

## Abstract

**Ethnopharmacological Relevance:**

In this study, we investigated the effects of* Tribulus terrestris* fruit (Leguminosae, Tribuli Fructus, TF) extract on oxazolone-induced atopic dermatitis in mice.

**Materials and Methods:**

TF extract was prepared with 30% ethanol as solvent. The 1% TF extract with or without 0.1% HC was applied to the back skin daily for 24 days.

**Results:**

1% TF extract with 0.1% HC improved AD symptoms and reduced TEWL and symptom scores in AD mice. 1% TF extract with 0.1% HC inhibited skin inflammation through decrease in inflammatory cells infiltration as well as inhibition of Orai-1 expression in skin tissues. TF extract inhibited Orai-1 activity in Orai-1-STIM1 cooverexpressing HEK293T cells but increased TRPV3 activity in TRPV3-overexpressing HEK293T cells. TF extract decreased *β*-hexosaminidase release in RBL-2H3 cells.

**Conclusions:**

The present study demonstrates that the topical application of TF extract improves skin inflammation in AD mice, and the mechanism for this effect appears to be related to the modulation of calcium channels and mast cell activation. This outcome suggests that the combination of TF and steroids could be a more effective and safe approach for AD treatment.

## 1. Introduction

Atopic dermatitis (AD) is a chronic pruritic inflammatory skin disease caused by abnormal skin barrier function and aberrant immune responses along with cutaneous hyperreactivity to environmental triggers [1]. AD has a complex etiology that involves activation of multiple immunological and inflammatory pathways along with disruption of epidermal barriers, elevated IgE levels, peripheral eosinophilia, and a predominance of Th2 cells expressing IL-4, IL-5, and IL-13 [2]. The main treatment for AD is skin hydration with emollients and suppression of cutaneous inflammation using topical steroids to reduce the number and severity of flares [1]. However, the use of steroids should be limited to the most severe cases due to their side effects, which include adrenal suppression, osteoporosis, hypertension, diabetes, obesity, and striae [3]. Recently, new treatments for AD, such as Th2 antagonists, cytokine antagonists, phosphodiesterase inhibitors, barrier repair therapies, and allergen-directed immunotherapy, have emerged. Although these therapeutic agents have greatly improved patient outcomes, the current treatments for AD are still not ideal and novel therapeutic strategies are required in the search for better drugs with safety and efficacy. 

Increasing interest in various medicinal plants and their bioactive ingredients has led to studies for AD treatment. The fruits from* Tribulus terrestris* L (Tribuli Fructus: TF, family Leguminosae) have various uses in traditional medicine, including pacifying the liver, depression relief, tonify blood and dispel wind, improving vision, and itch treatment as well as treating dizziness, insomnia, phlegm nodule, scrofula, and goiters [4]. TF is also used in folk medicine for a tonic, aphrodisiac, palliative, astringent, stomachic, antihypertensive, diuretic, lithontriptic, and urinary anti-infective agents [5]. In modern experimental studies, TF has been reported to have protective effects against liver and kidney toxicity [6–9], antidiabetic effects [8], and antioxidant effects [10]. This plant contains known bioactive compounds, such as cytoprotective lignanamides [10], anthelminthic [11] and antifungal saponin [12], and anti-inflammatory N-trans-*ρ*-caffeoyl tyramine [13].

In traditional Korean medicine (TKM), TF is used for improving eye trouble, as well as liver and kidney disorders, cutaneous pruritus, edema, inflammation, and tracheitis in skin diseases [13]. Furthermore, TF has been prescribed for the treatment of skin inflammation in AD. Nevertheless, the effects of TF on AD and its mechanism of action require clarification. In this study, we investigated the effects of TF extract on skin inflammation in an AD animal model and also investigated the mechanism responsible for the effects of TF extract in AD on the modulation of calcium channels and the activation of mast cells. In addition, we evaluated the possibility of combined treatment with TF extract and steroid for the development of more effective and safe therapies. For this, we conducted an* in vivo* study through application of TF extract and/or hydrocortisone in low dose.

## 2. Materials and Methods

### 2.1. Plant Materials and Preparation of TF Extract

Dried fruits of* T. terrestris *(TF) were purchased from the Medicinal Materials Company (Omniherb, Kyungsan, Korea). TF was authenticated by Professor Y.-K. Park, a medical botanist in the Department of Herbology, College of Korean Medicine, Dongguk University (DUCOM). Voucher specimens were deposited in the Herbarium of the DUCOM under registration number 2016-TF-E30. TF (200 g) were extracted with 2 L of 30% ethanol for 3 h, filtered through Whatman Grade 1 filter paper (Sigma-Aldrich, St Louis, MO, USA), concentrated under a vacuum rotary evaporator at 60°C, and then lyophilized in a freeze-dryer at −80°C with 5 mTORR (Il Shin BioBase Co., Yangju, Korea). The obtained TF power (yield of 8.8%) was stored at 4°C until use, at which time it was dissolved in a propylene glycol (Sigma-Aldrich).

### 2.2. Animals

Experimental 5-week-old SKH-1 hairless mice (20 ± 1 g, female) were purchased from Raonbio Co. (Yongin, Republic of Korea). All mice were maintained under constant conditions at 23 ± 2°C and 55 ± 5% humidity with free access to food and water. After acclimation for 1 week, a 12 h day/night cycle was maintained. The mice were cared for and used following the animal welfare guidelines issued by the Institutional Animal Care and Use Committee (IACUC) at Dongguk University (IACUC-2015-07126).

### 2.3. Preparation of AD Mouse Model

**Figure 1:**
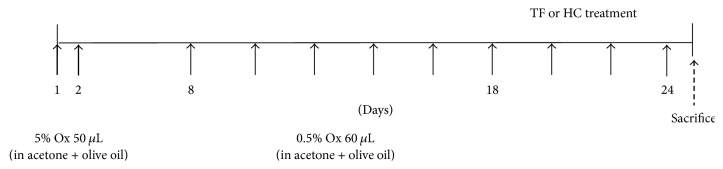
Experimental schematic showing preparation of the AD mouse model.

After 1 week of acclimation, the back skin of all animals was shaved using clippers and 8 mice were put into each group, including the normal group, AD-induced control group, 1% TF extract-applied group, 1% TF extract with 0.1% hydrocortisone- (HC-) applied group, and 1% HC-applied group as a positive control. The back skin of all groups, except the normal group, was treated with 50 *μ*L of 5% oxazolone (Ox, Sigma-Aldrich) dissolved in acetone and olive oil (4 : 1) on days 1 and 2 and then sensitized with 60 *μ*L of 0.5% Ox twice a day from days 8 to 24 (Figure 1). During sensitization, 200 *μ*L of 1% TF extract, 1% TF plus 0.1% hydrocortisone (HC), or 1% HC was applied to the same part once a day. On day 25, all mice were sacrificed and blood and skin tissues were harvested for analysis.

### 2.4. Evaluation of Transepidermal Water Loss

On day 25, transepidermal water loss (TEWL) was measured in three different parts of the dorsal skin using a TEWL machine (Vapometer, Delfin Technologies Ltd., Finland). Three different regions of the dorsal skin were measured for 10 seconds.

### 2.5. Evaluation of Symptom Scores

On day 25, symptom scores were measured in all mice as follows: 0, no symptoms; 1, mild (dryness, scaling); 2, moderate (dryness, scaling, and erosion); 3, middle (dryness, scaling, erosion, and excoriation); and 4, severe (dryness, scaling, erosion, excoriation, and hemorrhage). Three evaluators were blinded and separately evaluated the symptoms of each mouse and an average value for each group was obtained from the three scores.

### 2.6. Histological Analysis of Skin Tissues

After measuring symptom scores and TWEL, all mice were deeply anesthetized with isoflurane and sacrificed and back skin tissues were isolated. The tissues were fixed in 4% paraformaldehyde, and paraffin-formatted tissue blocks were made. Skin sections were cut into 3 *μ*m thick sections using a microtome and stained with hematoxylin and eosin (H&E) as well as toluidine blue. All stained tissues were observed by a microscope (Leica Co., Wetzlar, Germany). Eosinophils in H&E-stained tissues and mast cells in toluidine blue-stained tissues were counted in three different parts by blind observation.

### 2.7. Immunohistochemistry of Skin Tissues

Skin tissue sections were deparaffinized in xylene and dehydrated in graded alcohol. After washing the samples in PBS, sections were placed in epitope-retrieval buffer (DakoCytomation, Carpenteria, CA, USA) at 95°C for 20 min and subsequently cooled to room temperature (RT) for an additional 20 min. The sections were blocked with 10% goat serum in PBS, followed by blocking for endogenous peroxidases stained with peroxidase block solution (DakoCytomation). Sections were incubated overnight at 4°C with antibodies against anti-CD3 and anti-Orai-1 (Cell Signaling Co., Danvers, USA). Unbound antibodies were removed the following day by washing the slides three times with PBS. Areas positive for CD3 and Orai-1 induction were stained brown after development with diaminobenzidine. The slides were counterstained with filtered Mayer's hematoxylin (Sigma-Aldrich, St. Louis, MO), rinsed with distilled water, allowed to dry, and then mounted for viewing purposes. The images were observed using a Leica digital camera and microscope (Leica Co., Wetzlar, Germany).

### 2.8. Cell Culture

HEK293T cells and RBL-2H3 mast cells (ATCC, Manassas, VA, USA) were cultured in Dulbecco's modified Eagle's medium (DMEM) containing 10% fetal bovine serum (FBS) and 1% penicillin-streptomycin (Life Technologies, Carlsbad, CA, USA). For stable transfection of HEK293T cells with TRPV3, 10 *μ*g/mL of blasticidin (Thermo Fisher Scientific, Waltham, MA, USA) was added for antibiotic selection. HEK293T cells were grown at 37°C in a humidified incubator with 10% CO_2_/20% O_2_. RBL-2H3 cells were grown at 37°C in a humidified 5% CO_2_ incubator.

### 2.9. hSTIM1 and hOrai-1 Transfection

The cDNAs encoding human Orai-1 (hOrai-1) and human STIM1 (hSTIM1) were purchased from OriGene Technologies (Rockville, MD, USA) and then subcloned into pcDNA3.1 according to the manufacturer's protocol (Life Technologies). Human TRPV3 (pReceiver-M02) was purchased from Genecopoeia (Rockville, MD, USA).

For the electrophysiological experiments, HEK293T cells were seeded in 35 mm^2^ culture dishes (Thermo Fisher Scientific, Waltham, MA, USA) 1 day before transfection. The cells were transfected three times with hSTIM1, hOrai-1, and pEGFP-N1 using Turbofect™ transfection reagent (Thermo Fisher Scientific) according to the manufacturer's instructions. Transfected cells were selected under a patch clamp system; that is, cells showing green fluorescence due to expression of green fluorescent protein in pEGFP-N1 were selected using fluorescence microscopy. To record Orai-1 currents, hOrai-1, hSTIM1, and pEGFP-N1 were transfected at a ratio of 4.5 : 4.5 : 1. Experiments were performed after 24 h of transfection.

### 2.10. Electrophysiology

Patch pipettes were pulled using borosilicate thin wall glass capillaries (World Precision Instruments, Sarasota, FL, USA) in five stages using a programmable horizontal Flaming/Brown style micropipette puller (Model P-1000; Shutter Instruments, Novato, CA, USA). Pulled pipette tips were fire-polished using a microforger (Narishige, Setagaya, Tokyo, Japan) to 2.5–3 MΩ when they were filled with an internal solution and immersed in an extracellular solution. Transfected HEK293T cells were transferred into a perfusion chamber (Warner Instruments, Hamden, CT, USA) mounted on the stage of an inverted microscope (Nikon, Tokyo, Japan). Current through the cell membrane was recorded using conventional whole-cell patch clamp methods. Data were acquired using an Axopatch 700B amplifier (Molecular Devices, Sunnyvale, CA, USA) and digitalized using a Digidata 1440A (Molecular Devices) set at 10 kHz. To reduce electrical noise, data were filtered through a low-pass filter at 5 kHz using pCLAMP 10.4 software (Molecular Devices). Extracellular solutions were perfused by a gravity-driven perfusion system at a concentration of 3 mL/min. TF extract and chemicals were diluted into extracellular solution to the desired final concentrations and applied to the cell through the perfusion system. Liquid junction potentials were adjusted to zero before gigaseal formation. After the whole-cell configuration was established, cell capacitances were measured and compensated for electronically using an Axopatch 700B amplifier. To measure TRPV3 currents, voltage clamp protocols were applied every 20 s from −100 mV to 100 mV over 100 ms. Holding potential was adjusted to 0 mV. For hOrai-1 current measurement, ramp-like pulses from −130 mV to 70 mV over 100 ms were applied every 30 s at a holding potential of −10 mV. All voltage and current trace data were saved on a desktop computer and analyzed using Clampfit 10.4, Prism 6.0 (GraphPad, La Jolla, CA, USA), and Origin 8.0 (Microcal, Northampton, MA, USA). All experiments were performed at room temperature (23–25°C).

### 2.11. Experimental Solution for Whole-Cell Patch Clamp Study

To measure TRPV3 current, a pipette solution was prepared containing 140 mM CsCl, 10 mM EGTA, 4.85 mM CaCl_2_, 3 mM MgATP, and 10 mM HEPES adjusted to pH 7.2 with CsOH. The extracellular solution for TRPV3 was prepared and contained 139 mM NaCl, 5 mM KCl, 10 mM HEPES, 3 mM BaCl_2_, 2 mM MgCl_2_, 1 mM EGTA, and 10 mM glucose adjusted to pH 7.4 with NaOH. To measure Orai-1 current, the pipette solution was prepared with 130 mM Cs-glutamate, 20 mM 1,2-bis(O-aminophenoxy)ethane-N,N,N′,N′-tetraacetic acid, 1 mM MgCl_2_, 3 mM MgATP, 0.002 mM sodium pyruvate, and 20 mM HEPES adjusted to pH 7.2, and the extracellular solution was prepared with 135 mM NaCl, 3.6 mM KCl, 1 mM MgCl_2_, 10 mM CaCl_2_, 5 mM D-glucose, and 10 mM HEPES adjusted to pH 7.4. To activate the Orai-1 current, 20 *μ*M inositol 1,4,5,-triphosphate (IP_3_), which can deplete endoplasmic reticulum (ER) Ca^2+^ stores, was added to the pipette solution before the experiments.

### 2.12. *β*-Hexosaminidase Release Assay

A colorimetric assay was performed to determine *β*-hexosaminidase activity in RBL-2H3 cells using a *β*-N-acetyl glucosaminidase (NAG) activity assay kit (Biovision, Zurich, Switzerland). Cell culture supernatant (70 *μ*L) was incubated with 5 *μ*L of 5 mM substrate solution (5 mM p-nitrophenyl-N-acetyl-*β*-D-glucosaminidase dissolved in 0.2 M sodium citrate buffer, pH 4.5) at 37°C for 0.5 h. The enzyme reaction was terminated by adding 25 *μ*L of stop solution (0.1 M Na_2_CO_3_/NaHCO_3_, pH 10.0), and the absorbance was measured at 400 nm with a microplate reader (OASYS, Seoul, Korea).

### 2.13. HPLC Analysis

Standard solution was prepared by dissolving a rutin standard in methanol (0.1 mg/mL), and TF extract was dissolved in methanol (50 mg/mL) by sonication for 30 min at room temperature. The solution was centrifuged at 10,000 rpm for 5 min and then filtered with PVDF (0.45 *μ*m). HPLC analysis was performed using a Prominence HPLC system (Shimadzu, Kyoto, Japan) equipped with two pumps (LC-20AD), an autoinjector (SIL-20A), a UV-detector (SPD-20A), and a column oven (CTO-20A). An Optimapak C18 column (250 × 4.6 mm, 5 *μ*m, RsTech, Korea) was used at 35°C for separation. The mobile phase was made up with 2% glacial acetic acid in water (A) and acetonitrile (B). All samples were eluted at a 1.0 mL/min flow rate, and the gradient elution conditions were as follows: 10% B at baseline, a linear increase to 15% B from 0 to 20 min, then to 85% B from 20 to 40 min, and then holding at 85% B from 40 to 50 min. Between the injection of samples, the system was equilibrated for 15 min at 10% B. Detection wavelength was set at UV 254 nm. Then 20 *μ*L of volume of all sample solutions was injected using an autoinjector.

### 2.14. Statistical Analysis

All experimental data were expressed as mean ± standard deviation (SD) by Graphpad Prism 5.0 (GraphPad Software, La Jolla, CA, USA). Comparison of each group was carried out by Student's *t*-test and one-way ANOVA, and *P* < 0.05 was considered to be statistically significant.

## 3. Results

### 3.1. Effects of TF Extract on AD Skin Symptoms

**Figure 2:**
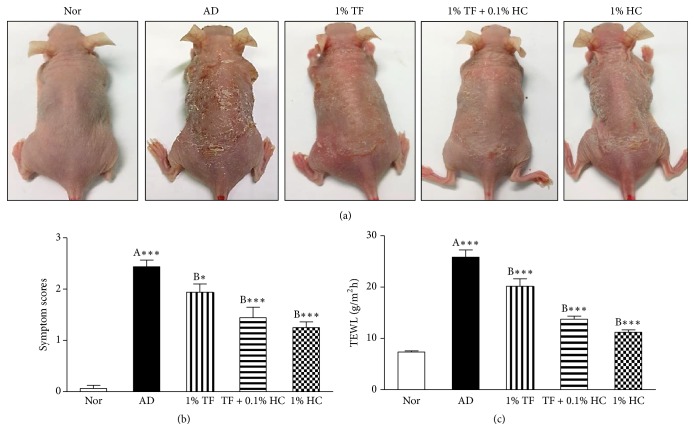
Effects of TF extract on symptoms and transepidermal water loss in oxazolone-induced AD mice. TF extract and HC dissolved in propylene glycol were applied to the dorsal skin of oxazolone-induced AD mice once a day from days 18 to 24. AD-like symptoms were examined with the naked eye and representative features were recorded (a). Symptom scores were measured in all mice as follows: 0, no symptoms; 1, mild (dryness, scaling); 2, moderate (dryness, scaling, and erosion); 3, middle (dryness, scaling, erosion, and excoriation); and 4, severe (dryness, scaling, erosion, excoriation, and hemorrhage) (b). TEWL was measured in three different parts of the dorsal skin with a Vapometer for 10 seconds (c). The results are expressed as the mean ± SD (*n* = 8 per a group). ^*∗*^*P* < 0.05 and ^*∗∗∗*^*P* < 0.001 versus normal (A) or AD control (B) mice. Nor, normal group; AD, oxazolone-induced AD group; 1% TF, 1% TF-applied group in AD control; 1% TF + 0.1% HC, 1% TF, and 0.1% HC-applied group in AD control; and 1% HC, 1% HC-applied group in AD control (*n* = 8 per a group).

To investigate the effects of TF extract on AD skin symptoms, symptom scores were measured in the dorsal skin of AD mice using a scoring index. As shown in Figure 2(a), AD symptoms such as dryness, scaling, erosion, excoriation, and hemorrhaging were observed in the dorsal skin of AD control mice and an application of 1% TF extract without or with 0.1% HC improved AD symptoms. Symptom scores were significantly elevated in the AD control group (*P* < 0.001) compared to the normal group. An application of 1% TF extract with 0.1% HC on the dorsal skin of AD mice significantly (*P* < 0.001) reduced symptom scores compared to the control group (Figure 2(b)). Application of 1% HC also significantly reduced symptom scores (*P* < 0.001), but there was no significant difference in the 1% TF extract only group.

To investigate the skin moisturizing effects of TF extract, we next measured TWEL in the dorsal skin of AD mice. In our results, water loss was significantly elevated in the AD control group (*P* < 0.001) compared to the normal group (Figure 2(c)). Application of 1% TF extract with or without 0.1% HC in the dorsal skin of AD mice significantly (*P* < 0.001, respectively) inhibited water loss compared to the control group. Application of 1% HC also significantly inhibited water loss (*P* < 0.001) in AD mice.

### 3.2. Effects of TF Extract on Histological Changes in Skin Tissues

**Figure 3:**
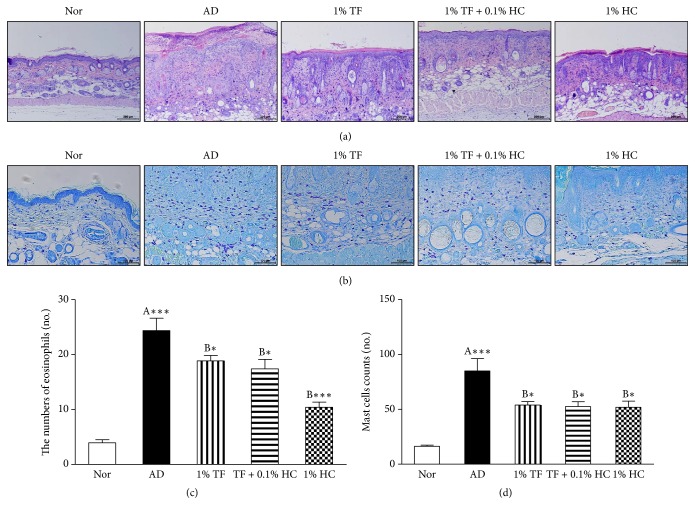
Effects of TF extract on histopathological changes in the skin tissues of oxazolone-induced AD mice. Dorsal skin tissues were stained with H&E (a) or Toluidine blue (b) and observed under a microscope (×100 for H&E, ×200 for toluidine blue). Eosinophils (c) and mast cells (d) were counted in epidermal regions. The results are expressed as the mean ± SD (*n* = 8 per a group). ^*∗*^*P* < 0.05 and ^*∗∗∗*^*P* < 0.001 versus normal (A) or the AD control (B) group.

To investigate the effects of TF extract on skin inflammation, histological changes in dorsal skin tissues were observed by H&E and toluidine blue staining. H&E staining confirmed a thicker dermis region along with infiltration of inflammatory cells in the oxazolone-induced AD control group (Figure 3(a)). Application of 1% TF extract improved this histopathological feature and significantly (*P* < 0.001) reduced the numbers of eosinophils, which were typical inflammatory cells, in the epidermal region (Figure 3(c)). Application of 1% TF extract with 0.1% HC or 0.1% HC alone also significantly inhibited infiltration of eosinophils. Toluidine blue staining confirmed that mast cells were significantly (*P* < 0.001) elevated in the AD control and vehicle groups (Figure 3(b)). Application of 1% TF extract with or without 0.1% HC significantly reduced mast cell numbers (*P* < 0.05, resp.), and application of 1% HC significantly inhibited mast cell infiltration into skin tissues (Figure 3(d)).

### 3.3. Effects of TF Extract on Infiltration of CD3^+^ T Cells and Expression of Orai-1 in Skin Tissues

**Figure 4:**
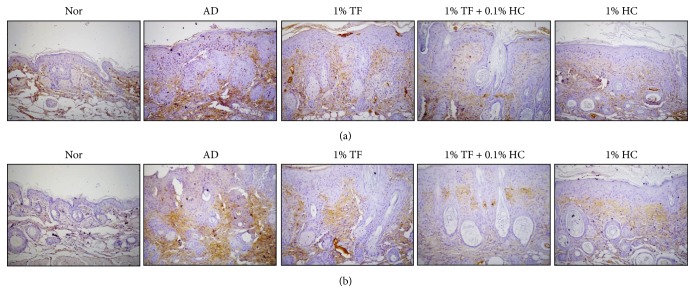
Effects of TF extract on infiltration of CD3+ T cells and expression of Orai-1 in the skin tissues of oxazolone-induced AD mice. Dorsal skin tissues were stained with anti-CD3 (a) or anti-Orai-1 (b) antibodies and then observed under a microscope (×100). Brown color, CD3+ T cells or Orai-1-expressing cells.

Next, we measured T cell infiltration into skin tissues after AD induction as well as expression of Orai-1 as a major ion channel, which induces skin barrier dysfunction, using immunohistochemistry. Expression of CD3^+^ T cells in skin tissues was elevated along with epidermal thickness in the AD control mice and was reduced by application of 1% TF extract with or without 0.1% HC (Figure 4(a)). Expression of Orai-1 was also elevated in AD mice and reduced by application of 1% TF extract with or without 0.1% HC (Figure 4(b)).

### 3.4. Effects of TF Extract on Activation of Orai-1 and TRPV3

**Figure 5:**
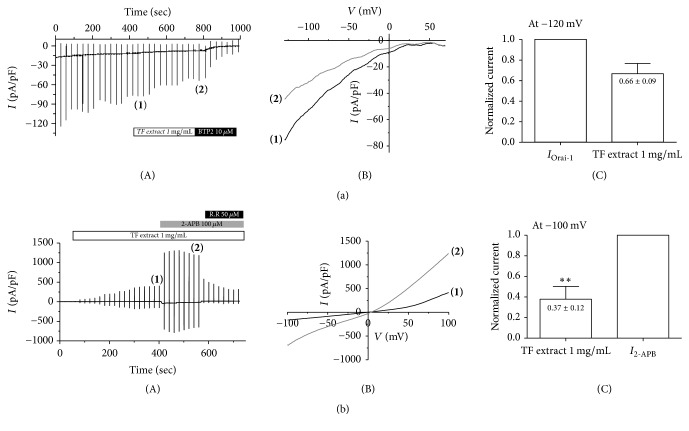
Effects of TF extract on calcium channels in HEK293T cells. Orai-1 current (*I*_Orai-1_) in HEK293T cells coexpressing Orai-1/STIM1 (a). Representative chart trace recordings of *I*_Orai-1_ currents (A). The number of parentheses represents steady state *I*_Orai-1_ (1) and its inhibition by 1 mg/mL of TF extract (2). Related* I*-*V* relationships of (1) and of (2) in (b). Summary of inhibition rate of *I*_ORAI1_ by TF extract at −120 mV (C). The results are expressed as the mean ± SD (*n* = 3). ^*∗*^*P* < 0.05 versus *I*_Orai-1_. Effects of TF extract on activation of TRPV3 in HEK293T cells overexpressing TRPV3 (b). Representative chart recording of *I*_TRPV3_ activation by TF extract (A). The number of parentheses represents steady state TRPV3 current (*I*_TRPV3_) after TF extract treatment (1) and the maximal current induced by 50 *μ*M 2-APB (*I*_2-APB_) (2). Related current-voltage (*I*-*V*) relationship curve at (1) and (2) from Figure 6(b). Normalized graph of *I*_TRPV3_ activation by TF extract and 2-APB at −100 mV (C). The results are expressed as the mean ± SD (*n* = 3). ^*∗∗*^*P* < 0.01 versus *I*_2-APB_.

To determine the modulatory effects of TF on ion channel activation in CD4^+^ T lymphocytes, we performed a whole-cell patch clamp study of Orai-1 calcium channels, which can generate intracellular calcium signaling via T cell receptor stimulation to activate T cells (Figure 5(a)). The Orai-1 channel was activated by endoplasmic reticulum (ER) calcium store depletion via direct coupling with the STIM1 protein, which is an ER Ca^2+^ sensor (Ref). Therefore, we cotransfected Orai-1 and STIM1 into HEK293T cells. To induce ER Ca^2+^ store depletion, we added 20 mM 1,2-bis(o-aminophenoxy) ethane-N,N,N′,N′-tetraacetic acid (BAPTA), a strong Ca^2+^ chelator, and 20 *μ*M IP_3_ to the pipette solution. After a membrane break-in, IP_3_ slowly diffused to the cytoplasmic side and stimulated the ER IP_3_ receptor. Eventually, depletion of ER Ca^2+^ stores slowly generated an inward rectifying current, *I*_Orai-1_ (Figure 5(a)(A)). After confirming the steady state *I*_Orai1_ (Figure 5(a)(B)), we perfused 1 mg/mL of TF extract (30% EtOH) into the bath solution. Treatment with TF extract significantly inhibited *I*_Orai-1_ by 34 ± 0.09% compared to control currents (Figure 5(a)(C)). At the end of the experiment, we treated an Orai-1 specific blocker, 2-aminoethoxydiphenyl borate (2-APB), to confirm its basal current (Figure 5(a)(A)).

Next, we investigated whether TF extract also activated TRPV3 channels, which are related to skin barrier formation via transglutaminase activation (Figure 5(b)). After observing no basal current under control conditions, we added 1 mg/mL of TF extract (30% EtOH) to the bath solution in HEK293T cells stably expressing TRPV3. After confirming steady state *I*_TRPV3_ through TF extract treatment, we treated 2-APB (>98% purity), which is a TRPV3 agonist, to confirm maximal current and add 50 *μ*M ruthenium red, which is an inhibitor of TRPV3 (Figure 5(b)(A), (B)). Treatment with 1 mg/mL of TF extract significantly (*P* < 0.01) increased *I*_TRPV3_ activation to 37 ± 0.12% (−100 mV) compared to 2-APB-treated current (*I*_2-APB_) (Figure 5(b)(C)). These results indicate that TF extract can reduce skin barrier impairment through activation of TRPV3 ion channels.

### 3.5. Effects of TF Extract on Mast Cell Degranulation

**Figure 6:**
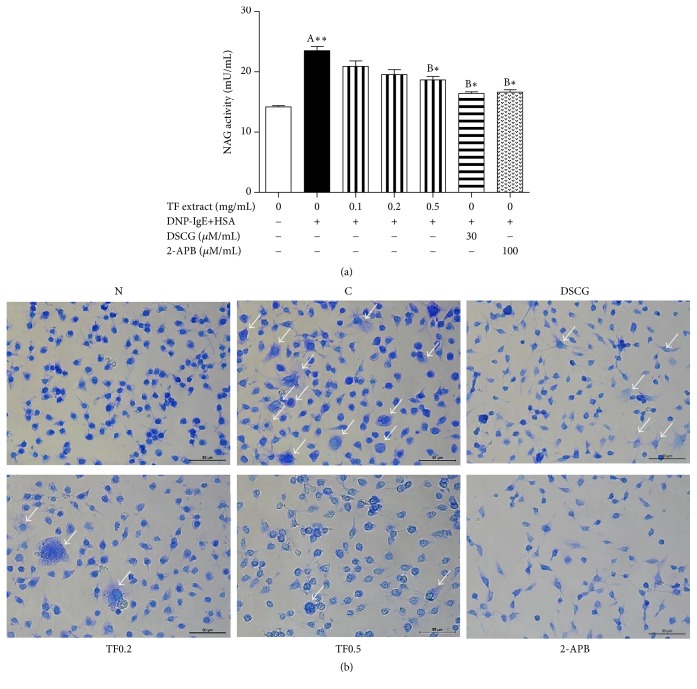
Effects of TF extract on *β*-hexosaminidase activity and degranulation in IgE/Ag-stimulated RBL-2H3 cells. The cells were stimulated with anti-DNP-IgE (0.1 *μ*g/mL) for 24 hr, treated with TF extract (0.1, 0.2, and 0.5 mg/mL), DSCG (30 *μ*M/mL), or 2-APB (100 *μ*M/mL) for 1 hr, and then stimulated with DNP-HAS (5 *μ*g/mL) for 3 hr. *β*-Hexosaminidase activity was measured in culture supernatants using an NAG activity assay kit (a). All data are expressed as the mean ± SD (*n* = 3). ^*∗*^*P* < 0.05 and ^*∗∗*^*P* < 0.01 versus normal (A) or DNP-IgE control (B) cells. The cells were stained with toluidine blue and morphological changes were observed under a microscope (×200) (b). The arrows indicate the degranulated cells. N, normal cells; C, DNP-IgE+HSA-stimulated cells; DSCG, DSCG-treated cells; TF0.2, TF extract (0.2 mg/mL)-treated cells; TF0.5, TF extract (0.5 mg/mL)-treated cells; and 2-APB, 2-APB-treated cells.

To investigate the inhibitory effects of TF extract on mast cell degranulation mediated via inhibition of ion channels, such as Orai-1 and TRPV3, *β*-hexosaminidase activity was measured as a biomarker of degranulation. Release of *β*-hexosaminidase from IgE-antigen (Ag) complex-stimulated RBL-2H3 mast cells was significantly elevated compared to unstimulated cells (Figure 6(a)). Pretreatment with TF extract at concentrations of 0.1, 0.2, and 0.5 mg/mL significantly suppressed degranulation of IgE-Ag complex-stimulated cells in a dose-dependent manner. In addition, treatment with TF extract reduced degranulation of RBL-2H3 cells stimulated with IgE/Ag (Figure 6(b)). Furthermore, release of *β*-hexosaminidase from IgE/Ag-stimulated RBL-2H3 cells was significantly inhibited by treatment with disodium cromoglycate (DSCG), which is a mast cell stabilizer, and 2-APB. No cytotoxicity was observed in the MTT assay at any concentration of TF extract when the cells were incubated for 24 h (data not shown). These results suggest that TF extract may function as a mast cell stabilizer by inhibiting IgE-Ag complex-mediated degranulation.

### 3.6. HPLC Analysis of TF Extract

**Figure 7:**
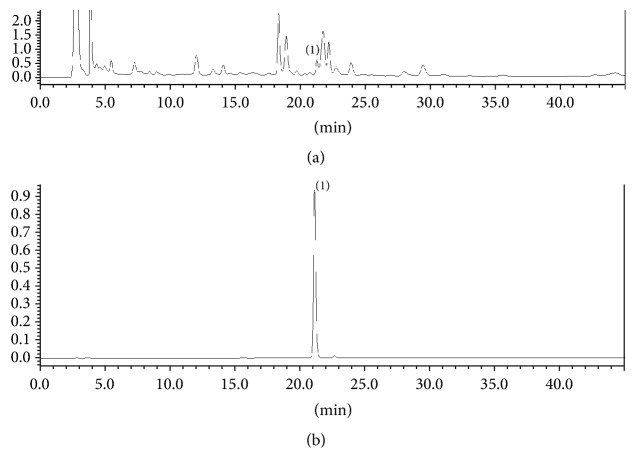
HPLC chromatogram of TF extract (a) and rutin (b) standard analyzed on an Optimapak C18 column with a gradient elution of mobile phases of 2% glacial acetic acid in water and acetonitrile along with wavelength detection at UV 254 nm. Peak identification: (1) rutin.

HPLC analysis was performed to quantify the rutin content in TF extract as a marker compound. As shown in Figure 7, rutin appeared at 21.2 min. The calibration curve of rutin in the ranges of 1.0 to 25.0 *μ*g/mL showed a linear regression coefficient of 0.9998. The rutin content in the TF extract was 0.03%.

## 4. Discussion

AD is a chronic inflammatory skin disease, and the number of patients with AD is increasing worldwide [14]. Topical steroids are currently the most common AD treatment, and HC is commonly used to treat skin inflammation caused by numerous conditions, such as allergic reactions, eczema, or psoriasis [1]. Although initial AD therapy is typically based on 1% HC, new effective therapeutic regimens are needed due to common side effects, such as skin redness, burning, itching, peeling, nausea, heartburn, headache, dizziness, and insomnia.

Herbal therapy for skin disorders has been used in traditional medicine for thousands of years, and specific herbs have recently been used as new therapeutic materials [15, 16]. TF is an herbal medicine used for the treatment of cutaneous pruritus, edema, inflammation, and tracheitis in TKM [8]. In terms of the herbal characteristics, this herb has a mild temper as well as a pungent and bitter taste. Its target organ is the liver, and it is effective for the control of headaches, dizziness, breast lumps, chest pain, flank pain, intestinal pain, and problems with breast milk circulation. Modern pharmacological studies have detailed its various effects against diabetes [8] and liver and kidney toxicity [7] as well as oxidative damage [10], but its herbal features and clinical efficacy are not well known. Therefore, in this study, we investigated the efficacy of TF extract with or without 0.1% HC to improve AD using an Ox-induced AD mice compared to 1% HC application as a common topical dosage.

AD is a relapsing chronic inflammatory disease in the skin characterized by rash, pruritus, eczema, and xerosis through abnormal inflammatory and hyperimmunological pathways [17, 18]. These pathways include excessive infiltration of inflammatory cells, such as macrophages, lymphocytes, eosinophils, and mast cells, infiltrating skin lesions, as well as a high level of serum IgE [2]. The skin barrier has two key functions, including prevention of excessive water loss and blockage of harmful substances, such as irritants and allergens from the environment [19]. Therefore, disruption of the skin barrier increases TEWL via reduction of stratum corneum hydration and causes penetration of harmful substances, which results in opportunistic secondary infections [20]. Thus, this defective inside-outside skin barrier presents typical AD symptoms, such as dryness, scaling, erosion, excoriation, and hemorrhaging. Accumulation of inflammatory cells in the AD skin barrier is easily observed and induces production of inflammatory cytokines, which leads to skin barrier remodeling with increased epidermal and dermis thickness as well as accumulation of inflammatory cytokines. In particular, mast cells play a key role in AD pathogenesis and are activated by cross-linking of the high affinity IgE receptor (Fc*ε*RI) and B cell-producing IgE, which results in the release of Th2 cytokines, IL-4, and IL-13, which induce phenotypic symptoms, such as the IgM to IgE switch, fibrosis, epithelial hyperplasia, and barrier dysfunction [2, 21]. Eosinophils also contribute to skin barrier remodeling through production of Th2 cytokines, IL-4, and IL-5. In our study, TF extract improved skin symptoms in Ox-induced AD mice via reduction of TWEL and inhibition of inflammatory cell infiltration, such as eosinophils and mast cells, which suggests that TF extract can protect the skin barrier against water loss and atopic inflammation. We also confirmed the inhibitory effects of TF extract on mast cell degranulation by measuring *β*-hexosaminidase release along with morphological changes. These results suggest that TF extract application to the skin of AD mice can improve symptoms through regulation of mast cell-mediated allergic responses and skin inflammation.

Since the introduction of Nc/Nga mice as a spontaneously occurring model of AD, several other AD mouse models induced by skin injury and epicutaneous sensitization with allergens, such as ovalbumin, house dust mites, or hapten, have been developed over the past two decades [22, 23]. Ox is a heterocyclic compound and is used for development of AD model [24]. When applied to the skin of hairless mice, the mice developed AD symptoms, such as barrier dysfunction, secretion of IgE and Th2 cytokines, hyperplasia of epithelial cells, fibrosis, and infiltration of inflammatory cells into the dermis and epidermis [25]. In this study, we observed that skin symptoms in Ox-induced AD mice were improved by application of 1% TF extract or a combination of 0.1% HC in low dose, which was similar to the effects of 1% HC, and that improvement suggested that TF extract could prevent skin barrier destruction. Meanwhile, we did not observe changes in IgE and Th2 cytokine levels (data not shown), which suggested that our model included the acute AD phase. Several studies have reported that an Ox challenge for 3 weeks in mice could not induce Th1 and Th2 cytokine production in serum but did lead to cytokine production in ear or spleen tissues [26–28]. Therefore, they recommended a longer challenge period for* in vivo* study of the induction of serum IgE or Th2 cytokines.

Recently, investigations into ion channels, such as Orai-1, TRPV1, and TRPV3, have shed new light on potential targets for the treatment of inflammatory skin diseases, such as AD. These ion channels have been shown to directly modulate epidermal proliferation, differentiation, barrier homeostasis, and inflammation in the skin [29]. Ca^2+^ influx through these channels eventually generated intracellular Ca^2+^ signaling, which resulted in different outcomes depending on the individual Ca^2+^ channel type, such as lymphocyte activation through Orai-1 [30], epidermal barrier formation and keratinocyte differentiation through TRPV3 [31] and itch generation through TRPV1 [32]. Therefore, a specific agonist/antagonist for each calcium channel is required for maintenance of skin barrier homeostasis and for treatment of dermatological diseases, such as AD. Therefore, Orai-1 and TRPV3 could be potential targets for the treatment of AD. Recently, as a part of our ongoing research to find ion channel-modulating medicinal plants from natural sources, we found that the application of 1% TF extract with or without 0.1% HC reduced Orai-1 expression and infiltration of CD3^+^ T cell into skin tissues. BTP2 is a potent inhibitor of Orai-1 channel which regulates the activation of immunocytes such as T lymphocytes. BTP2 decreases store-operated Ca2+ entry (SOCE) mediated by Orai-1 channels. Subsequently, Ca2+ dependent functional responses such as the activation of calcineurin and cytokine production are suppressed in lymphocytes [33, 34]. 2-APB is a common activator of TRPV channels such as TRPV1, TRPV2, and TRPV3 [35]. Ruthenium red not only blocks TRPV3 but also inhibits TRP channels in a broad-spectrum specifically [36]. In our* in vitro* study, treatment with TF extract resulted in significant inhibition of Orai-1 activation and induction of TRPV3 activation. These findings suggest that TF extract can reduce skin barrier dysfunction associated with allergic inflammation in AD through modulation of the ion channels Orai-1 and TRPV3. In our previous study, we found the regulatory effects of TF methanol extract on Orai-1 and TRPV3 calcium channels [37].

We detected rutin as a main compound in TF extract via HPLC analysis. The suppressive effects of rutin on AD and allergic contact dermatitis have been reported [38]. However, further studies are required to investigate the recovery mechanism of TF extract in skin barrier dysfunction through modulation of calcium channels to understand the therapeutic effects of TF extract and rutin on AD. Recently, the genotoxic effect of TF extract at the high concentrations in cultured peripheral human lymphocytes has been reported [39]. Although in vitro study, this should be taken into consideration for the development of new herbal medicines using TF extract.

## 5. Conclusions

We observed that application of 1% TF extract or a combination of TF extract with 0.1% HC to the skin of oxazolone-induced AD had beneficial effects on skin barrier function, had been protected against excessive water loss, and had inhibited inflammation by blocking infiltration of inflammatory cells, such as T cells and eosinophils. The working mechanisms affect modulation of the calcium channels Orai-1 and TRPV3, as well as inhibiting mast cell activation. This result suggests that TF extract can be used as a natural source for the development of new medicines to reduce toxicity induced by topical steroids and increase efficacy through a combination with steroids. To the best of our knowledge, this study is the first to investigate whether herbal medicines can be use in a combined treatment with Western medicines for AD.

## Abbreviations

AD: Atopic dermatitis
H&E: Hematoxylin and eosin
HC: Hydrocortisone
TF: Tribuli Fructus
TEWL: Transepidermal water loss
TRPV3: Transient receptor potential vanilloid subtype 3.
